# The Teeth and Their Treatment

**Published:** 1884-08

**Authors:** Elton R. Smilie

**Affiliations:** San Francisco, Cal.


THE
AMERICAN JOURNAL
OF
DENTAL SCIENCE.

VOL. XVIII. THIRD SERIES—AUGUST, 1884. No. 4.

ARTICLE I.

### THE TEETH AND THEIR TREATMENT.

BY ELTON R. SMILIE, M. D., SAN FRANCISCO, CAL.

The structural condition as lesions of the teeth not only affords a certain index to the habits and customs of the present possessors, but reflect back ancestral, through an indefinite series of generations. Even favorable indications interposed and continued for a few successive birth scores leave improved impressions which cannot be concealed from the observing dentist of quick perceptions, notwithstanding neglect and direct injury have again resumed their sway. To the physician who devotes his attention to the treatment of chronic diseases, these hereditary impressions offer a far better means for the detection of characteristic taint than the patient's rehearsal of symptoms can give, except of those from recent acute accession. In ratio with the grade of material, and perfection of union in the structural affinites of the teeth, correct judgement can be formed of the deviations of the osseous system from a normal state; although, in fact, there is no reflex action, and the constituent elements of the teeth differ in composition and density of cohesive arrangements from the flesh-covered bones of the body. The development of the teeth from the pulp expansion of the nerve vessels and neurotic sheath, through which the formative supply of material is derived, shows clearly in the varied elements of the deciduous and permanent sets a special secretive offset from the ossfic laboratory designed for their production. The type of this vicarious functional offset is readily recognized in the nacre excretive formations of the oyster and kindred mollusks, for self protection, in addition to subserving the purposes of teeth. This alien adoption and its object is demonstrated in the partiform exposure and adaptation of the crowns for the mill purposes of attribution in grinding food for special fluid admixture in furtherance of digestion. The pulpy and other conditions of the infant at birth would aptly suggest that in original formation the human system became a body corporate by a united organic communism for combined generative propagation in sexual intercourse. This would seem to admit of proof from conchoidal resemblance, as there are perfect shell representations of all the external organs of sense and reproduction that belong to the human body, while the inhabitants in organic likeness enact in isolated counterpart the functions which would distinguish them as representative organisms of the human system. The eleboration, characteristic elements, and formative adoption of the teeth by the jaws, directly imitate, in pulp excretion, the shell formation of the oyster. *The deciduous teeth* in normal coaptive development with the osseous models of the infantile jaws, practically exemplify in constructive material, and class, forms designed for special service in the varied process requirements of mastication, the first stage of a provisional economy intended to fulfill at maturity, functional demands in the preparation of food for an omnivorous organic community. If the deciduous pulp is examined with magnified power in the process of development, its oyster-like simplicity will appear in strong contrast with the secreting membranous folds designed for the combined excretion of the enamel-nacre, cementum, and osteo-dentine, which, in adaptative reduction, become the highly vitalized centre and root sheaths of the permanent teeth. Yet, with the evident increase of vitalized power necessary for the elaboration of structural material requisite in laminated and concentric combination to sustain intact the relation of parts to resists erosive agencies and functional attribution, at maturity, the semi-alien alliance of the teeth with the animal body remains the same. With a perfectly normal predisposition the nacre, or "milk teeth," are excreted from, and modeled by, the pulp with an exact relation in size and order to correspond with osseous formation of the jaw, insuring regularity and structural conditions necessary for the healthy fulfillment of their limited period of service. The "fifth year teeth," when they emerge from the gum at the masseter angles of the jaws, represent a distinct era in child life, inasmuch that they foreshadow in structural formation the degree of perfection of the permanent set, while they retain in reflection the nacre impression of the deciduous. As the influence of mouth habits preserve or impair the natural integrity of transmissible material impressions, the artificial deteriorations practiced for the creation of morbid appetites by the past and present civilized generations, have in train caused diseased constitutional defects, which have entailed upon the infant in utero a pathological inheritance that in teething reflects back to its mouth source. When the normal conditions of a natural birthright are fulfilled, the bony ossification at the apex of the jaws proceeds in pari passu development with the nacre teeth, and when they are matured afford, at the "cutting" period, a firm fulcrum support for their easy exit through the gum integument. In the same manner, and like exactness, the second teeth oppose their cutting edges and grinding surfaces to the points of the deciduous roots, and by absorptive pressure supplant and subextract their crowns with anæsthetic freedom from pain. But, with the insatiable habits begot from the narcotic stimulation of tobacco, malt, and other spirituous liquors, the exceptions to mal-formation, irregularity, painful dentition and rapid decay from the local renewal of cause, are in near approach to the zero of non-existence. As the suffering of the infant in teething are entailed with an enforced life, passion begot, in reckless disregard of cause and effect, they have an especial claim for the sympathetic study of the dentist. As in speech, taste, etc., the mouth adjuncts are indicators of remote bodily emotions and functional aberrations in more advanced life, so in infancy to the pulp developing teeth, and rudimentary jaw ossifications, may safely be referred, under existing evils, death, is the providential deliverer from an inherited existence of suffering. When the incipient teeth and jaw osssfications are lacking in well affinitized material necessary for perfect combination and mutual stimulus in the process of development, the assistance of the mother's fingers can be rendered serviceable, under directions, to supply the aid required by nature.

Even in the more matured state of the jaw in childhood, that precedes the appearance of the second incisors, mothers dutifully interested, and obedient children have, under the dentist's direction, averted irregularities by the stimulating use of their fingers in expanding them by pressure. When at fully matured puberty the wisdom teeth complete the sum total allotted to the series in dentition, they present in greater or less degrees of perfecting the crowning foundation for the exercise of the dentist's reparative art. Or if benevolently predisposed, with patrons inclined to reciprocate his interest in their welfare, ready to advise means adapted to structural peculiarities for the preservation of the teeth in freedom from decay.

THE PERMANENT TEETH.—In keeping with their semi-alien formation and adoption by the jaws are separators, comminutors, and grinders of food for organic nutriment, the teeth, in normal perfection, possess, as shown, structural adaptations for self preservation, in comparative freedom from the action of chemical agencies generated in the mouth and stomach, as well as from the wearing attrition of their cutting and crown surfaces in working contact.

By passing in review the more complex secreting and excreting conditions of the nerve vessels, neurotic sheath, and pulp expansions, required in the formation and adaptation of the permanent set to increase in growth of jaws to the terminal period, the structural limits of comparative insensibility from the acutely sensitive, will be readily recognized, and render clear the adaptability of materials and methods of adjustment necessary to be employed for safely fulfilling the preservative resources of the dental vocation. As appears in formative process, the excretive pulp acts as a model to each class of teeth in quadruple correspondence, reflecting from side to side of each jaw peculiar indentations and defects; although, from some local cause the secreting membranes often fail to preserve the same structural unity in the supply and material affinites of composition.

The outer membrane is the secreting and excreting medium of the nacre-enamel, the required fibrine, etc., being supplied from self-absorption in the process of crystalization, the quality of each being determined by the hereditary normality of the general system.

As the structural formation reveals, the depositing membrane of the cementum is involved with that of the nacre-enamel in the line of distinction, uniting the outer semi-crystaline with the inner fibro-laminated, embracing in combination the resisting and non-conducting qualities required for the protection of the more elastic and sensitive dentine in its connection with the vitalized pulp. These two stage processes of model formation are affected from below, independent of the osseous radii maturing for the alveolar socket enclosure of the root. Seemingly, after these oyster functions of the pulp have excreted and formed the protective shell, destined to be exposed to the vagaries of artificial habits cultivated by human beings, it enters into, a n osteo-alliance with the secretive functions of the jaws producing dentine, which extends its crown-covered body downwards and forms the roots from internal secretion, within the duplication of its outer fold, which, with the completion of the ossific process, forms the alveolar lining membrane of the root and socket. The dentine invests and forms the pulp cell, and from continued functional attachment is known to deceive from it reparative aid in kind, when exposed.

The "pulp" is the highly vitalized centre, and is the excreting formative source of the structural elements entering into the varied combinations of tooth substance, acting as the terminal constructive expansion of the neurotic sheath and nerve vessels for the transmission of material; its functional duties cease with the death of the body, but is much more frequently terminated by the broach and forceps of the dentist.

DENTAL FALLACIES.—The same emperical infatuation has retarded the preservative progress of operative dentistry, that has attended the general and special practice of medicine since their first professional inauguration as checks to disease. Yet, from their semi-alliance with the human body, and peculiarities of structure promised in adaptation to functions that required insensibility to force and grinding attrition in contact, the teeth present certain indications necessary to be followed for their preservation in freedom from decay, of which use and cleanliness are the chief. When decayed, and the dentist's service is required for their repair, it would appear legitimate that he should understand the bearings of their relation, and extent of functional reciprocation that they hold, as instruments of service, to the organs of the human system, in order to secure a successful result to his labors. From the aid of preliminary observations founded upon the experience of self sensation, in comparison with the teeth of his patients, he would be able to detect the cause of structural deficiencies, and from the knowledge obtained would be able to judged correctly of the methods taught for the arrest of decay, and thus, step by step, within the range of self approved security, would make his progress certain. The oyster-like simplicity of formation in structural combination, with well defined uniting lines of demarcation between the nacro-chrystaline enamel, which is free from elasticity, and cementum—which is the ivory of teeth—and possesses kindred affinities, with elasticity and its comparatively non-conducting qualities, acting as an intermediate shield for the protection of the more highly organized dentine which remains in connection with the pulp, the teeth would seem to offer a chart of direction for the reparative art of the dentist bordering upon the infallible. Especially, as there is no reflex functional neuralgia from pulp irritation, and inflammation, as the local influences of disease are imparted from alveolar contact of the teeth with the jaws. The pioneer dentists of fifty years ago were obliged to rely chiefly upon their own test experience, as there was little recorded anterior to serve as a guide to their operations. This dearth necessitated extreme caution and practical forethought, so that there were but few missteps that required regretful reconsideration. In filling—the main source of income to the dentist—they used only hand pressure, the more fastidious giving a surface finish with slight touches of the mallet; even the faint jar it caused was a source of dread to the patient; and in cavities that reached the dentine the hand of the operator rarely ventured its use, from fear of ulceration.

As a guard against the excessive flow of saliva, their sole reliance was placed upon the use of the napkin, and instead of water injected from a syringe, to act as a detersive for the removal of decayed matter, the secretions ejected from the glands served the purpose.

In addition to the primitive cleanliness of the means, the modern dentists, with proclivities for fashionable empiricism, have often expressed in print their wonder at finding the walls of the cavity perfectly free from decay, after having removed a slightly impacted filling of ten or more years' service, to be replaced by a "first-class" substitute, engine welded, within the rubber screen of a coffer-dam. If they had, even by comparative analysis, tested the effect of water in the cavity of a tooth prepared for filling, in contrast with the *peculiar* antiseptic qualities of the various glandular secretions thrown into the mouth for food commixture, they would have found in water the active elements for the continued decay of the tooth substance, while in opposition the saliva possesses the inmate essentials of retardation· Aside from the constituent resemblance to the gland fluids of the mouth to those of the nacre secretions of the oyster, the relish the latter affords as the most congenial of carnivorous edibles, is proof presumptive of a natural alliance in the formative and preservative conditions of the teeth The frequent injection of water into the cavity of a tooth in the preparatory process of excavation must, of necessity, leave an impression of dampness, however careful the operator may be in the use of absorptives and actual heat for evaporation; and as its constituents are the active source of fermentative decomposition in vegetable and animal matter, and of reactive oridization, in a greater or less degree to mineral, the effects likely to be produced by a metallic substance in contact with diseased dentine can be readily anticipated, in process, by an analytic mind.

Or if his experience has failed to note the impression of water in reaction upon dentine in contact with a metallic filling, in contrast with saliva, the experiment would prove satisfactorily to the dentist, and more especially to his patient. The non-conducting properties of saliva in opposition to water can be made apparent by placing a dossil of cotton wet with each, alternately, in the cavity of a sensitive tooth; or by immersing extracted teeth, with the dentine exposed, in saliva and water separately, the contrasted effects can be tested; but the saliva must be free from contamination for the development of its conservative properties. For cleaning the teeth, water is indicated, as it removes adhesive matter from the enamel, and allows the preservative qualities of the saliva to come in direct contact with the surface. We know, from self observation, that when hungry, the odor of cooking food excites, in anticipation of its relish, a flow of saliva, and that gradually, as our appetite becomes appeased, it decreases in proportion, and if eating is persisted in, when the gland stimulation ceases, *as the sure sign of enough*, the teeth become dry and cumbered, and deglutition difficult. If these natural indications of limit in mouth service could be placed within the control of childen by education, the personalities of the community would be greatly benefitted in health and comfort, although the physician and dentist might suffer in pocket. *The Painful and Destructive Fallacy* of engine "plugging" is still more apparant to reasonable judgment and repugnant to natural indications, than the use of water as a detersive agent for removing excavated decayed matter from the dentine-exposed cavity. From the compactness and varied density of the three composite parts that enter into the structural formation of the teeth, the effect of the destructive agencies is different in action on each, but bears no resemblance in either to necrosis or other diseases that effect integument covered bones; yet, as with the chelonia carapace shell protections of the toes and fingers, are extremely liable to infect the bones when subject to ulceration. But if, from fore-knowledge, and as a providence for the retardation of prospective degeneration, the teeth had been designed to afford a season for repentance, it would exceed the constructive power of man to suggest an improvement. Even the carapace nail defenses of the toes and fingers show a wonderful adaptation of means to ends, in the firm elasticity of their composition and attachment for the effective accomplishment of intention as shields; but, if we consider the service required of the teeth for the healthy prolongation of vitality, their more complex provision for safety excites in a higher degree our admiration.

Still, with unreason, mans' invention often tends,
Beyond the useful, and in self-destruction ends;
The dentist with lack of reason o'er acts his art
When for engine mallet he thrusts the jaws apart.

Although the structure of the teeth, and method of adoption, admits of hard usage in the grinding service of their vocation, when slightly diseased the immunity from painful irritation is lessened; but even in superficial cavities from fissure tendency to decay, which penetrate but little below the surface of the cementum, the safe course is to use as little force in excavation and filling as is consistent with through preparation for the permanent adjustment of the material used to arrest decay. The causes ot decay in teeth must, of necessity, vary in degree, the intensity of irritation, and render them more or less susceptible to external impressions, and the treatment should vary in accordance with structural imperfection and indications of the exciting cause of decomposition. Upon the adoption of this course for the cultivation of reason and judgment depends the dentist's merits for the professional distinction of doctor; but if he regards a tooth cavity as a hole to be filled with gold, welded by the strokes of a machine mallet, and finished up to class rules, heedless of vital alliance and regulation of force, the title of tinker would better distinguish his characteristics of worth. For the development of worth and support of professional dignity, collateral literature should be cultivated as a criterion of thought capacity. A lack of this, the only means of opening to public view the resources of mind, has reduced to the grade of empiricism the educated of the medical profession; as modesty and scorn will withhold their tongues from the cajoleries practiced in self praise by the charletan.

By the practice of thought discipline to interest, and raise the standard of public intelligence, there will be a mutual reaction for the benefit of each, both in professional and social intercourse, that will prove a source of reliance and stimulus to mental exertion on the part of the dentist. To the "crumb item," condensed, and scrap opinions, is mainly due to the diversity of materials and methods adopted in dental practice to obtain uncertain results. For a long period dentists complained that gold balled or curled under the blows of the "plugger," and the gold beaters tried all their metallurgical resources to render it in preparation pure, soft and pliant; but, with their almost perfect success, it was impossible to make it fulfill dental expectations when driven with the quick blows of their engine trip-hammers against elastic walls of dentine. Then came the wail, that gold-filled lateral cavities having a dentine base and walls in relative proportion to depth, would decay around the filling, "no matter how well condensed it might be;" and, without the slightest reference to cause, various suggestions were offered to remedy the defect; and at the present time "first-class fillings" of the description frequently have a gutta-percha base, with side patches of amalgam, all the work of the same dentist. Without other knowledge than an intutive perception of physics, the cause should have been apparent from the demonstration; the gold "balling" and curling upward around the points of the "plugger" from the quick blows in recoil, bespoke with mathematical certainty the impossibility of making semi-plastic material adapt itself in close contact with the elastic walls by the process.

In second consideration, his acquired knowledge should have taught him that organized dentine, when irritated, became an erectile tissue, and if imflammation and ulceration of the pulp did not succeed, shrinkage would, leaving an inevitable space between his "first-class filling" and walls of the cavities. If, in addition, he would examine his engine work with a critical eye, aided by magnifying power, he would discover in the grade closeness of adaptation the varied density of substance that enters into the composition of the teeth, from the clear lines of impressed demarcation caused by the degrees of structural elasticity between the nacre-enamel, cementum and dentine. A farther investigation of the connection existing between the dentine and pulp would enable him to calculate the probable chances of the latter's inflammation and ulceration from the effects of engine concussion.

It is well known that the pearl oyster (*Aviculidæ*) can detect the first attack of his enemy the teredo aviculœ upon his shell, which gives him time to deposit and unite an effectual wall of pearl-nacre to intercept the course of its bore, and as it is impenetrable to the forcep cutting jaws of this sea worm, it forms a safe barrier in the direct line of its intended entrance ; but as foilded appetite begets a corresponding sagacity to overcome obstacles opposed to its gratification, in every grade of animal life, from the poly-podo-vegetable to man, the boarer commences a sinuous course beneath the soft phosphate on the surface of its nacre attachment, untill he finds a thin and penetrable portion of the shell, through which he makes a quick entrance and secures his prey. Then the oyster, as in the death flurry of the whale, looses all command over its resources of plastic defense, and while struggling in death agonies from the gnawings of its voracious foe, yields himself, with its life, "contoured" into pearls the most beautiful symbols of soul existence that it is possible to conceive, with the sole exception of a comely woman's teeth when they reflect in purity the "angelic" mind of their possessor. This flurry was suggested in question whether the pulps of of human teeth have their oyster prototype's power of resisting "bacilla', and other vermiform species of invasion with reconstructive dentine ? There is certainly abundant testimony in proof that, with proper treatment, the excretive functions of the pulp can be revived in sufficient degree for the formation of a protective shield of dentine, but with a nearer resemblance to the cancellated structure of the jaw than the original, showing that there had been a change in the elementary secretion from disease, or that after the second set was completed the pulp adapted its functions to the osseous source of supply. Sixty years ago Dr. Harwood of Boston, recommended the use of cotton saturated in a melted combination of bayberry wax, two parts, to one each of creasote and oil of cloves, as a cap covering for exposed pulps ; and to this and similar applications we are indebted for the knowledge that with judicious protection they can form a substitute shield with the characteristics of dentine. But after the plunge of an engine bur, directed by careless hands, into the substance of a pulp, there is but a small change for its salvation, although slight wounds from the excavator heal readily. At the present day, within the attesting scope of a few correspondents, there are upwards of five hundred hand manipulated gold fillings, having a broad dentine base in near approach to the pulp, with various degrees of finish, from ragged to smooth, that have a painless service date ranging from twenty to fifty years; while in comparison, they acknowledge that there are but few engine-filled lateral cavities with a dentine base and walls that have eked out, with patching, a term of ten years. The invention and introduction of the Snow and Lewis automatic surface finishing hand instrument was a serviceable improvement over the hand mallet, inasmuch that it left a free hand to the operator, but its stroke was labored, and lacked in other essentials that would render it safe to use as an impactor within the boundaries of diseased dentine; yet, by a large proportion of those who express their judgment, unbiased by the vageries of advertising fashion, it is considered the best aid of its kind an advance of hand pressure.

"*Pivoting Teeth*" with wood demonstrates clearly the danger that is to be apprehended from the jar of the mallet; for, with the custom of striking home to its bearings a tooth with a close fitting pivot, ulceration of the root and alveolar caries were first introduced as an attendant upon that inconsiderate operations of the dentist. It was an expressed source of self-gratulation to Dr. Harwood, that never, to his knowledge, had a patient of his suffered from having a pivot tooth set, and he justly attributed the cause to his method. He used second-growth walnut wood, and prepared the size to suit by striking them through the grade holes in a screw plate, then immersed them in tincture muriate of iron until the pores were completely penetrated, and after they were perfectly dried with slow, artificial heat, subjected them to the action of equal parts of creasote and oil of cloves. In preparing a pivot, he obtained an exact fit, so that a junction could be obtained without other force than the use of his fingers, and in two instances reported, the teeth were firmly retained twenty years without being re-set. In none of the adjunct processes of the dental art, has there been so near an approach to letigimate perfection as in the manufacture of artificial teeth, by those who devote their attention to it as a specialty; but as in other departments, vaunts, gold medals, and fair diplomas, are not to be trusted as truthful tests of superior excellence.

Gold, and other fillings, although useful in the retardation of decay, are but blots that deface the architectual beauty of the mouth, and should act as beacons of warning to the human family, that the gratification of abnormal appetites begets like heedless passions, which entail in conception upon the resistless embryo, not only the structural defects of the teeth and their deformities, which have given birth to the dental profession, but a predisposition to all the diseases which effect the body corporate, which as guardians of the life sustaining portal, they act in the capacity of preparatory indexes.

*Woman's Fitness for the Dental Profession.*—There is but little doubt that with cultivation woman could develop all the essentials required for successful dental practitioners. But would the vocation prove congenial to the evident designs of nature instituted to govern the mutual division of labor responsibilities between the sexes? Already in America we observe the rapid ingrowth of familiarity between sexes, which is the offspring of foreign inoculation bred from an appropriate labor distinctions. Labor is not only a democratic equalizer, but it would prove a brutalizing source of degradation to the weaker sex if established upon a common grade. The proof of this axiom is demonstrated in the family relations of the German peasantry, both in the mind and matter of their personalities;—if the former can yield more than the mere supposition of its existence, as an attribute of mankind, rather than an effect from its actual impression. With the few ingrafts of women into professional life which have been accomplished, we can feel and foresee with its full realization, the utter annihilation of all the ideal charms of mother and home, the centres of an indwelling and enduring affection which clings to our memories as the ivy of hope to the latest thought and breath of life.

But as experience is the best practical and least heeded teacher of life, an example as an exception to common indifference will prove instructive, even if it lacks the full influence of persuasion.

*Extracts from a letter from Miss———, to her Dental Instructor:*

* * * RUSSIA, * *

MY DEAR AND HONORED TEACHER:—

I will not attempt to express the emotions of gratitude the reception of your letter caused, or the feelings of thankfulness for the strength which enabled me to overcome my prejudices against adopting an assumed name, as you advised, while practicing abroad a profession which necessity forced upon me as a choice. Above all the influence of your persuasion which made me limit my practice to the sole treatment of women and children. From novelty, as I am the first female dentist that ever practiced in the city, and some merit I hope, for the honor of your instruction, my success in every respect, has by far exceeded the utmost stretch of my imagination. My hands and arms gain daily in strength and skillful expertness in the use of my instruments for the adaptation of material for the preservation of natural, and supply of artificial, teeth. I have already two female students, or apprentices, of good families, and shall soon require the services of a third. But with all the pleasure that attends success, I cannot forget the ideal refinement of a home vocation, which is the heaven of hope to a pure women. As you advised, I have cultivated a dignified, but kindly disposed reticence, upon all subjects foreign to my profession, during "office hours"

(how odd the term sounds!) but have formed some acquaintances which when tested, may eventuate in friendship. The people are kindly disposed, but, according to our standard, lack delicacy and refinement. Visiting the Princess C——— a few evenings ago, her husband, first in joke, but afterwards in earnest, tried to induce me to receive him as a patient, and I anticipate, from his manner of treating my met hod o frefusal, future annoyance from others. *

You question the cause of the Russians' intolerance of the Jews; and seem to have had your sympathies aroused in their favor, as if they were blameless of provocation. But from what I have seen of them, I do not feel disposed to discredit the reports which charge them with acts that would put to shame those of their father Abraham, when he took his wife Sarah to Egypt and bartered her for service to the King of Gerar and others, under the pretence that she was his sister or daughter.

The Russians, or Sclaves, are not over-scrupulous in taxing self-restraint for the preservation of virtue; but it appears that even with them there is a limit to shameless prostitution that will barter the purity of daughters for a price in gold. All seem to rejoice in their forced exile, and wonder that the puritans of Yankee-land should receive them with sympathy. * * * * * I have discovered that the natural preference of Russia women is for male dentists, and the husbands *vice versa*, a choice that I am afraid will mar the prospects of female dental operators of my predilections throughout the civilized world; but I hope the novelty will last long enought to purchase my selfish desire for liberty. * * * There is one harbinger of good omen that promises a happy recompense for the minor evils of my exile; in visiting associations the conversation is often abruptly turned from general topics into a semi-philosophic discussion of the influences which most tend to enoble and elevate society, in the scale of mutual confidence and sympathy. Of course, with natural egotism peculiar to the sex, it was generally conceded that the cultivated womans' refinement and sincerity were influences absolutely necessary for the accomplishment of any good purpose; and as Prince C———,aptly remarked, the predenominating lack of those qualities gave the direct negative of brutality, which in reaction recoiled upon woman—as the conservative medium of example—with a downward power for her debasement; an illustration he cited the elevating influence of the English womans' dignified sincerity upon the natural characteristics, and in contrast from the opposite cause, German boorishness and vulgarity. This, and incidents of like import, afford me the tacid assurance of a gratifying appreciation, derived from exampled impression; and as it is the hope of my heart, I have no fear, as I am certain it will increase in power of expression from an approval that intimates the desire of adoption. * * * * * *

If all the females who select the profession of dentistry as the means of gaining a livelihood, from natural choice or that of necessity, should possess the quick perception and natural ability indicated in the above letter, we might hope that the influence of their exertions would tend to the discovery of a dental laboratory within themselves, which might, judicious co-operation on the part of male dentists, be made to reproduce natural sets of teeth without blemish, possessed of constituent materials derived from a healthy source, that would insure uniformity and life-long endurance. For the consummation of a result so desirable, it will, of course, be necessary to make the conceptive public *feel* that the transgression of nature's laws brings immediate suffering to the transgressor; as selfish gratification has become so predominant in democratic expression, a father of the herd, would scarcely abate a drink, cigar, or quid of tobacco to save his prospective child from the misery which their use, and like vitiated habits, are certain to entail. There was a time within the historical period of memories recollection, when the women of the United, states, without exception, would have esteemed it an insult for a man with a cigar or pipe in his mouth to offer his company to them on the street; at that period, the necessity for the service of a dentist was in so little demand his professional existence was confined to large cities. Until the introduction of lager beer and votaries to its corner grocery chapel of dispensation, whose ancestors had for centuries, in transmission, been inured to its degrading and stupefying effects, which from incorporation they consider a constituent part of themselves and entitled to citizenship, the teeth of the native Americans in regularity and freedom from decay bore testimony to the healthy simplicity of their habits. Colonization may be effected in a variety of ways, quite as repugnant to our natural feelings of honor and justice as when enforced by direct usurpation; and an undiscriminating toleration of emigrants, who bring with them habits of debasement sure to prove contagious and destructive to all the elements of kindly affection and refinement in association, as well as to the rational preservation of physical integrity, should at least be held in quarantine from citizenship, as examples of reproof, until they had given practical demonstration of a disposition to adapt themselves to the healthy regime of the country. The chlora and small-pox create alarm from epidemic invasion, but when the hitherto mysterious cause is removed, they leave in renewal a healthy impression; but the lager beer sot, and users of tobacco, are chronic examples of leprous habits, who deny the same in disposition nature's privilege of a atmosphere, and by breeding contagion, colonize its source, and deprive the well disposed of sustenance designed for the normal development of mind and body.

*Apsorptive Preservation of Decayed Teeth* may be effected by cotton or Chinese rice paper, frequently renewed in the cavity, without antiseptic addition, other than that peculiar to the retentive material and fluids of the glands. Numerous instances are cited—with proof authentication—in which exposed pulp had been treated by this simple method,—through fear from dental experience,—and preserved without pain, but with evident improvement in structural condition, for noted periods exceeding twenty years, the recuperative power of the pulps having supplied a substitute for, if not identical with, the original elements of dentine. As density adds to the conductive power of any material, the impaction should be regulated according to the sensitiveness of the exposed dentine and pulp; at first, the pressure should be just sufficient for the retention of the pledget, and if cautiously introduced, will, in a majority of cases, abate pain and absorb decayed matter from the inner surface of the cavity, as well as the food source of irritation from without. As timid persons usually prefer to trust to their own sense of feeling in the adjustment of remedial substances, a little instruction will enable them to accomplish it effectually; and dentists of clear judgment and long experience claim that the adoption of this method, is more satisfactory, in favorable cavities, for relieving pain and preparing the exposed dentine for contact with filling, than the free use of narcotico, as in reaction they often become irritants, and by causing expansion, make it necessary to remove the "plug" to prevent ulceration and alveolar abscess.

It has been clearly established by test observation and comparison, that diseased dentine which has been treated by the various obtunding preparations used by the dentist, and water for washing out the cavity, retains the moisture for months after extraction, while in those extracted without treatment the evaporation is much more rapid. As a natural sequence to cause and counter effect, engine-welded gold admits of no absorptive reaction, and, but for the inevitable space from the shrinkage of the dentine, after the irritation has subsided, few would escape the penalty of ulceration. Another effect that especially tends to weaken the cohesive tenacity of both cementum and enamel, is their deprivation of the peculiar vitality supplied from the dentine, by the unyielding pressure of the welded gold at the junction of each with the other, producing strangulation that prevents its transmission, causing them to crumble from attrition in mastication. The impression generally prevails that the absorptive tendency is from the mouth inward, but the contrary may be proved by testing the outer and inner surface of a filling, or pledged of cotton which has been for sometimes retained in the cavity of a tooth, as the latter, if closely impacted, will be found comparatively dry where it came in contact with the walls of the cavity, the glairy penetration of the saliva showing distinct traces of demarcation, but a few lines in depth from the surface.—*Dental Practitioner.*

